# An Evaluation Framework for Obesity Prevention Policy Interventions

**DOI:** 10.5888/pcd9.110322

**Published:** 2012-06-28

**Authors:** Jennifer Leeman, Janice Sommers, Maihan Vu, Jan Jernigan, Gayle Payne, Diane Thompson, Claire Heiser, Rosanne Farris, Alice Ammerman

**Affiliations:** Author Affiliations: Janice Sommers, Maihan Vu, Alice Ammerman, University of North Carolina at Chapel Hill, Chapel Hill, North Carolina; Jan Jernigan, Gayle Payne, Diane Thompson, Claire Heiser, Rosanne Farris, Division of Nutrition, Physical Activity, and Obesity, Centers for Disease Control and Prevention, Atlanta, Georgia.

## Abstract

As the emphasis on preventing obesity has grown, so have calls for interventions that extend beyond individual behaviors and address changes in environments and policies. Despite the need for policy action, little is known about policy approaches that are most effective at preventing obesity. The Centers for Disease Control and Prevention (CDC) and others are funding the implementation and evaluation of new obesity prevention policies, presenting a distinct opportunity to learn from these practice-based initiatives and build the body of evidence-based approaches. However, contributions from this policy activity are limited by the incomplete and inconsistent evaluation data collected on policy processes and outcomes. We present a framework developed by the CDC-funded Center of Excellence for Training and Research Translation that public health practitioners can use to evaluate policy interventions and identify the practice-based evidence needed to fill the gaps in effective policy approaches to obesity prevention.

## Introduction

The prevalence of obesity in the United States has led to increased investment in obesity prevention and a growing focus on interventions that extend beyond individual behaviors to also address changes in environments and policies (1,2). Individual-level interventions are resource-intensive and have limited potential for lasting success as long as environments promote unhealthy behaviors and limit access to healthy foods and safe opportunities for physical activity (3,4). Public policies at the federal, state, and local levels are among the primary mechanisms for changing environments (3,5) and can include, for example, transportation planning, nutrition standards, and federal food benefits (eg, Supplemental Nutrition Assistance Program [SNAP]) at farmers’ markets (6).

Despite the need for policy action to create healthier environments, little is known about policy approaches that are most effective (2,4,7). The Institute of Medicine (IOM) recently recommended responding to this challenge by taking advantage of “emerging and ongoing interventions as sources of practice-based evidence to fill the gaps in the best available evidence” (2). Funding from the Centers for Disease Control and Prevention (CDC) and others is spurring the emergence of promising obesity prevention initiatives, presenting a distinct opportunity to build the body of practice-based evidence. However, contributions from this policy activity are limited by incomplete and inconsistent evaluation of policy processes and outcomes. We present a framework for evaluating policy interventions that was developed by the CDC-funded Center of Excellence for Training and Research Translation (Center TRT) to build public health practitioners’ capacity to evaluate policy. The framework is designed for use by practitioners working as partners and evaluators in public policy initiatives (legislation, regulations, or funding allocations) at the state or local level.

## Background

Public health practitioners are central participants and leaders in the movement to implement public policies (6). Since 2004, the CDC’s Division of Nutrition, Physical Activity, and Obesity (DNPAO) has funded Center TRT to provide evidence and training to support public health practitioners working in obesity prevention (www.center-trt.org). To provide practitioners with a broad portfolio of interventions, Center TRT reviews and disseminates policy and environmental-change interventions that were developed and evaluated by either practitioners or researchers (8). By reviewing interventions, Center TRT staff have identified challenges in policy evaluation, such as identifying the start and end points of policy interventions, documenting processes and successes, and fully measuring success following policy enactment. In a recent Center TRT survey of practitioners (n = 82), the 2 most highly rated priorities for training pertained to evaluating policy interventions.

Interventions at the policy level require a different approach to evaluation than that used for interventions at the individual and group levels. Changing policy is a long, complex, and multistep process. Public health practitioners can work for years to raise awareness of a public health problem and potential solutions and, following enactment of new policy, can work still longer to assist with its full implementation. The process tends to be incremental and cyclical, involving the continual modification of existing policies and development of new policies. Among the most challenging aspects of the policy-making process are the influence of multiple sectors (eg, transportation industry, food industry) and contextual factors such as special interest groups, policy makers, and the broader sociopolitical environment (9,10). The involvement of multiple sectors requires that practitioners work with nontraditional partners; the influence of contextual factors confounds efforts to define the boundaries of a policy intervention. The challenge for public health practitioners, then, is collecting evaluation data on a process that is cyclical, incremental, and influenced by many factors outside their control.

## Center TRT’s Approach to Policy Evaluation

Center TRT’s approach to evaluating policy interventions is based on the use of emergent logic models, in other words, logic models that evolve as a project progresses (11). Logic models are a systematic and visual way to present the relationships among an initiative’s inputs, activities, outputs, and outcomes (12). The traditional approach to logic modeling involves the creation of a single model depicting a linear causal pathway from inputs to outcomes. This approach is difficult to apply to policy interventions because they involve a continual and often cyclical interplay among inputs, activities, and outputs and because the path to successful outcomes is neither linear nor constant (11,13,14). Practitioners can respond to this challenge by iteratively revising their logic model to fit progressive stages in the policy-making process to identify the emergence of new inputs, activities, and outputs over time (11).

**Figure 1:**
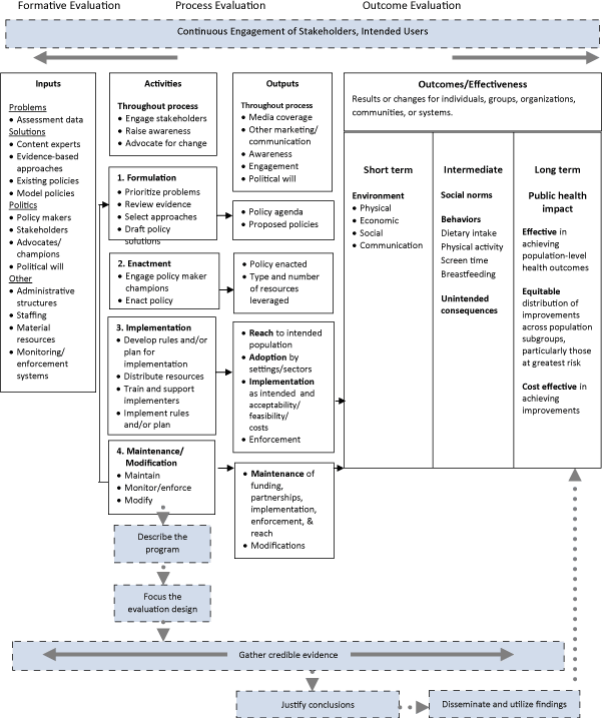
Center TRT’s evaluation framework incorporates elements from multiple policy-making and evaluation frameworks (9,15-19). The framework is intended to support practitioners as they develop logic models to describe and evaluate policy making initiatives. Shaded portions of the figure represent the Centers for Disease Control and Prevention’s Evaluation Framework (16).

The cornerstone of Center TRT’s approach to evaluation is its evaluation framework (Figure 1). The framework is intended to provide a comprehensive overview of the entire process with the expectation that practitioners will select and adapt those components that are relevant to the stage of their initiatives and use them to develop their own progressive logic models (11). Center TRT’s framework draws on a range of theories and frameworks related to policy making and evaluation in public health (Table). Foundational to the framework is CDC’s guidance on the 6 steps essential to all evaluations (Figure 1): continuously engaging stakeholders and intended users, describing the program, focusing the evaluation design, gathering credible evidence, justifying conclusions, and disseminating and using findings (15). The framework is organized according to the 4 standard sections of a logic model: inputs, activities, outputs, and outcomes (12). We describe these 4 sections in more detail and provide an illustration of how practitioners might apply them to evaluate a state-level farm-to-school policy to create infrastructure and allocate funding to coordinate the purchase and distribution of locally grown foods to schools.

**Table:** Overview of Frameworks and Theories Used in Center TRT Evaluation Framework

Framework or Theory	Central Constructs
CDC evaluation framework (15)	Steps in an evaluation: Engage stakeholders Describe the policy initiative Focus the evaluation Gather credible evidence Justify conclusions Disseminate and use findings Phases of evaluation: Formative Process Outcome

Process of setting the policy agenda – creating a window of opportunity (16)	Components of process: Problem Solutions to problems Politics

Process of making public policy in the United States (9)	Steps in policy making: Formulation Enactment Implementation Modification Sources of input and feedback: Organizations Interest groups Sociopolitical environment

RE-AIM framework (17)	Public health impact criteria: Reach Effectiveness Adoption Implementation Maintenance

Conceptual framework for environmental and policy strategies (18)	Types of environments: Physical Economic Social Communication

Criteria for evaluating health care systems (19)	Criteria: Effective Equitable Cost effective

Abbreviation: Center TRT, Center of Excellence for Training and Research Translation; CDC, Centers for Disease Control and Prevention.

### Inputs

*Inputs* are the resources and contextual factors that support and influence each step in the policy-making process. The selection of inputs to include in the framework was informed by Kingdon’s theory that 3 elements need to align to create a “window of opportunity” for new policy: problems, solutions to those problems, and politics (16). Using farm-to-school policy as an example, inputs related to the problem include assessment data on the prevalence and distribution of obesity and unhealthy eating behaviors among the state’s school-aged children. Inputs related to solutions include content experts, evidence-based approaches, existing state policies, and model policies such as exemplar farm-to-school policies enacted in other states. Politics are critical to whether a problem rises to the top of the political agenda and consist of the attitudes and activities of policy makers, stakeholder groups, advocates/champions, and the general public. Political inputs may include the extensiveness of farm-to-school activities at local levels and the attitudes of politicians, school staff, teachers, farmers, and health advocates.

After a policy is enacted, additional inputs include the administrative structures, staffing, and material resources necessary to implement the policy and the systems that will be employed for policy monitoring and enforcement. The systems in place to purchase foods and distribute them to schools or to monitor school districts’ implementation of existing policies are examples of these inputs.

### Activities

*Activities* are the actions or events that engage and transform inputs to produce outputs and outcomes. Engaging stakeholders, raising awareness, and advocating for change are essential activities throughout the policy-making process (9). Stakeholders are defined broadly to include special interest groups, the general public, policy makers, representatives from the sectors or settings that will implement the policy, and others with an investment in the policy and its outcomes. Additional activities in the framework are organized according to commonly recognized steps in the policy-making process: formulation, enactment, implementation, and maintenance/modification (9).

**Formulation**. Activities involved in formulating policy include reviewing evidence on the problem and potential solutions, gaining stakeholder agreement on priority problems and preferred approaches, and drafting policies in the form of laws, rules, and funding priorities (20).

**Enactment**. To enact a policy, activities focus on looking for windows of opportunity, identifying the policy makers who will sponsor or promote the policy, and formally enacting the drafted policy in the form of a law, regulation, or budget. Enacting farm-to-school policy, for example, might involve passing new legislation and allocating funding.

**Implementation**. Implementing the policy may include activity at multiple levels of the executive branch of government or by involved settings and sectors. For example, in implementing new state-level farm-to-school policy, the Department of Education may reallocate resources and draft new rules governing how the policy will be implemented across school districts. School districts and schools would then develop and implement plans and allocate the resources necessary to implement the new policy.

**Maintenance/modification**. At the final stage in the process, activities are directed toward maintaining policy implementation and ensuring sustained effect through monitoring, enforcement, and further policy modification. The framework includes a feedback loop from maintenance to formulation to indicate that policy making is cyclical; modifications strengthen policies over time.

### Outputs

*Outputs* are the direct, tangible results of activities. Outputs include media coverage and other communication (eg, policy briefs). They also may include evidence of increased stakeholders’ awareness of the problem, engagement in the policy-making process, and political will to take action.

The central outputs of the first 2 steps of policy making (formulation and enactment) are the actual policies proposed and enacted. Practitioners may assess the extent to which policies employ evidence-based approaches, in other words, approaches that have been found in prior research to be effective at improving health environments (2,10). Evaluation may also assess the extent to which policies followed model policy guidance from organizations with expertise in obesity prevention policy (eg, National Policy and Legal Analysis Network to Prevent Childhood Obesity). For example, does the farm-to-school policy include language that is strong enough to ensure the resources necessary to support implementation (5)?

Outputs of the second 2 steps of policy making (implementation and maintenance/modification) were drawn from elements of the RE-AIM framework. The RE-AIM framework is widely used to assess an intervention’s potential impact and includes the following criteria: whether the intervention reaches the priority population, is effective in achieving intended outcomes, is adopted by providers and settings, and is implemented with fidelity and maintained over time (17,21). The outputs of the implementation and maintenance steps of the framework address 4 of the 5 RE-AIM criteria: reach, adoption, implementation, and maintenance. The fifth criterion, effectiveness, is addressed in outcomes.

*Reach* is the absolute number, proportion, and representativeness of people exposed to the policy. For farm-to-school policy, people reached may include children, family, community members, school employees, and farmers. The policy could be evaluated on the basis of both the absolute number and the proportion of the state’s school-aged children affected. Reach also accounts for the extent to which affected children represent the state’s overall population of school-aged children, particularly those at disproportionate risk for obesity.

*Adoption*, in the case of voluntary policies, is the absolute number, proportion, and representativeness of settings/sectors that decide to participate or to implement the policy. For example, farm-to-school policies require that local farmers, food distribution systems, and schools agree to participate (ie, adopt). Adoption is less applicable to mandatory policies. Adoption can also apply to multiple levels of government, such as a local governing body’s decision to enact policy to operationalize state-level policy. For example, a state may enact enabling legislation in support of complete streets. County-level governments may then “adopt” the legislation by enacting local regulations.

*Implementation* is the extent to which a policy is applied as intended among levels, settings, and sectors. For example, implementing farm-to-school policy can occur at the state, school district, and local school levels. Additional outputs include acceptability and affordability. Evaluation of farm-to-school implementation might address whether locally grown foods were delivered to schools and served in cafeterias as intended; cafeteria staff, parent, and student response (ie, acceptability); and the effects on the cost of school lunches (ie, affordability).

*Maintenance/modification* involves assessing continuation of funding, partnerships, implementation, and reach over time. For farm-to-school policies, outputs include whether schools continue to order and serve the same or greater amounts of locally grown foods as when the program started. Policies may also need to be modified. For example, municipalities may have restrictions on foods served in school cafeterias; policies may need to be modified to allow schools to serve foods grown in school gardens.

### Outcomes

*Outcomes* are the desired and unanticipated results of a policy. Short-term outcomes are changes to the environment that promote healthier foods and increase physical activity. Changes can occur in the following types of environments (18):

physical (eg, proximity to healthier food and spaces for physical activity)
economic (eg, changes to prices, taxes)
social (eg, changes to social networks)
communication (eg, advertisements, point-of-decision prompts).

An increase in the amount of fruits and vegetables available in school cafeterias is an example of a short-term outcome of farm-to-school policy.

Intermediate outcomes refer to behavior changes that occur as a result of a policy’s effects on environments and include changes in dietary intake, physical activity, screen time, and breastfeeding. Intermediate outcomes also refer to changes in social norms related to obesity and the behaviors that prevent it (18). Thus, intermediate outcomes of a farm-to-school policy may include changes to school children’s attitudes toward and consumption of fruits and vegetables.

Practitioners cannot anticipate all possible consequences and, therefore, should assess both positive and negative unintended outcomes (22). For example, incorporating local foods into unhealthy recipes is a potential unintended consequence of farm-to-school policies.

The long-term goal of obesity prevention policy is to be effective, equitable, and cost-effective at the population level (19). The policy should have the potential to contribute effectively to improvements that are distributed equitably across subgroups to reduce disparities in obesity and obesity-related health outcomes (2). Policies also should be cost-effective; in other words, they should use resources in ways that contribute to improvements that are equal to or greater than alternative policies or programs.

Although the framework outlines short-term, intermediate, and long-term outcomes, many initiatives will capture change only in short-term outcomes (ie, changes to the environment). CDC guidance on community-level obesity prevention identifies a range of measures that can be used to assess environmental change (23). Changing behaviors and health outcomes at the population level will require multiple initiatives and an extended period and will, therefore, often exceed the scope of any single initiative (2).

## Discussion

**Figure 2:**
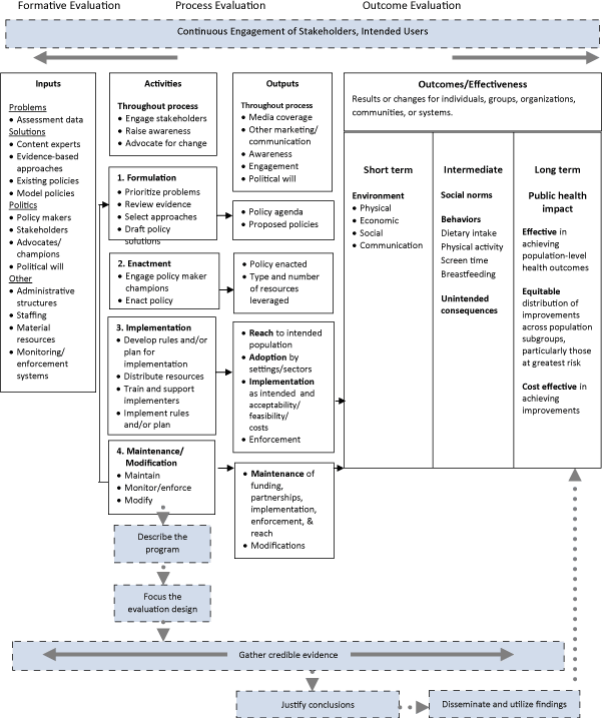
The emergent logic model presents inputs, activities, outputs, and outcomes that might be included in a farm-to-school policy initiative during the formulation stage of the policy-making process. Solid lines depict components that apply to the current state of policy. Dotted lines depict potential future activities, outputs, and outcomes.

Practitioners can draw on the Center TRT framework to identify and adapt components to include in their progressive logic models. For example, practitioners who are evaluating the formulation of a policy will identify inputs such as data needed to assess political will, develop the policy, and identify stakeholders (formative evaluation). They will then identify activities and outputs that are relevant to formulating policy such as engaging stakeholders, raising awareness, and drafting policy solutions (process evaluation). Although it is premature to measure outcomes at the stage of policy formulation, practitioners still should identify the intended outcomes, which may change as the process evolves. In addition to revising logic models as they progress through the policy-making process, practitioners also should revise them in response to changes in elected officials and in the broader economic and social environment (9). Figure 2 provides an illustration of a logic model for a farm-to-school policy that is in the formulating stage of policy making.

Once a logic model has been created, practitioners can use it as a guide to developing evaluation questions and an evaluation plan. Center TRT has created a list of evaluation questions and potential data sources that are mapped to components of the framework (available by request to corresponding author). The list includes sections related to formative, process, and outcomes evaluation.

The CDC’s evaluation framework calls for the continuous engagement of stakeholders, which can be challenging in obesity prevention because it often involves diverse sectors and disciplines (2). Center TRT’s framework can facilitate engagement by providing a tool that helps practitioners visually summarize a policy initiative and collaboratively identify outcomes of importance to each stakeholder. Identifying processes and outputs during policy formulation may be important to retaining stakeholder engagement before policy enactment. Furthermore, evaluation findings are critical to identifying and addressing areas in need of improvement. Findings can, for example, help to distinguish whether disappointing outcomes are due to problems with the policy or with its implementation.

The framework has limitations. Although it begins to capture the complex and emergent nature of policy making, the framework may yield logic models that oversimplify. Policies interact with other existing and emerging policies to affect outcomes; activities and outputs also may be designed to address the requirements of multiple policies. Further development is needed to assess how well the framework accommodates these types of real-world complexity. Center TRT staff are beginning to use the framework to provide evaluation technical assistance to practitioners and to develop logic models and evaluation plans for the policies Center TRT disseminates. To fully assess the framework’s utility, we need to evaluate practitioners’ perceptions of its usability and to study its effects on the quality of policy evaluations.

Obesity is a threat to the health of the US population. Nationwide, communities and states are responding to the challenge by implementing a range of innovative policy approaches. Public health practitioners can help evaluate these emerging interventions and contribute the base of “best available evidence” (24). The proposed framework has potential to support practitioners’ efforts to evaluate policy by promoting the creation of emergent logic models and adapting logic models components to fit the distinct needs of policy evaluation.
